# Intraoperative optical coherence tomography guided corneal sweeping for removal of remnant Interface fluid during ultra-thin Descemet stripping automated endothelial keratoplasty

**DOI:** 10.1186/s12886-021-01934-2

**Published:** 2021-04-15

**Authors:** Michael Mimouni, Martin Kronschläger, Manuel Ruiss, Oliver Findl

**Affiliations:** 1 Department of Ophthalmology, Rambam Health Care Campus, Haifa affiliated with the Bruce and Ruth Rappaport Faculty of Medicine, Technion-Israel Institute of Technology, Haifa, Israel; 2 Department of Ophthalmology and Vision Sciences, University of Toronto, Toronto, Ontario Canada; 3 Vienna Institute for Research in Ocular Surgery (VIROS), a Karl Landsteiner Institute, Hanusch Hospital, Heinrich-Collin-Strasse 30, AT–1140 Vienna, Austria

**Keywords:** Intraoperative optical coherence tomography, Interface fluid, UT-DSAEK, Graft thickness, Sweeping

## Abstract

**Background:**

Remnant interface fluid following Descemet stripping automated endothelial keratoplasty (DSAEK) is associated with postoperative detachments. The aim of this study was to assess outcomes of intraoperative optical coherence tomography (iOCT) guided meticulous peripheral corneal sweeping for removal of interface fluid during ultra-thin (UT) DSAEK.

**Methods:**

This retrospective study included all eyes underwent iOCT guided UT-DSAEK from October 2016 to February 2018 at the Hanusch Hospital, Vienna, Austria. Peripheral meticulous corneal sweeping was performed to remove excess fluid. Central graft thickness (CGT) was measured prior to surgery, after graft bubbling and after corneal sweeping. Remnant interface fluid rates were compared between eyes that underwent rebubbling and those that did not.

**Results:**

Overall, 28 eyes of 28 patients with a mean age of 73.9 ± 10.0 years were included. An iOCT guided meticulous peripheral sweeping was performed in 89.3% (*n* = 25) of the cases. Following 84% (*n* = 21) of the peripheral sweeping performed, remnant fluid was no longer identified. Following peripheral sweeping the interface fluid height was reduced from 17.31 ± 15.96 μm to 3.46 ± 9.52 μm (*p* < 0.001) and CGT was reduced by 7% (*p* < 0.001). Rebubbling was performed in 17.9% (*n* = 5) of the cases. The rebubbling group had a greater proportion of patients that had remnant fluid identified with iOCT at the end of surgery despite meticulous peripheral sweeping (60.0% versus 4.4%, *p* = 0.01).

**Conclusion:**

The iOCT identified subclinical remnant fluid in nearly 90% of UT-DSAEK cases. An iOCT guided peripheral corneal sweeping led to resolution of interface fluid in a majority of cases. Eyes with persistent remnant fluid despite peripheral corneal sweeping are more likely to require subsequent rebubbling.

**Supplementary Information:**

The online version contains supplementary material available at 10.1186/s12886-021-01934-2.

## Background

Partial thickness corneal transplants currently account for a majority of keratoplasty procedures performed [1, 2]. Descemet stripping automated endothelial keratoplasty (DSAEK) and Descemet membrane endothelial keratoplasty (DMEK) are considered the procedures of choice for corneal endothelial decompensation [3]. Endothelial keratoplasty leads to faster visual recovery, fewer complications, superior visual outcomes and lower graft rejection rates compared to penetrating keratoplasty (PKP) [4].

Despite its relatively high safety profile, DSAEK is not without potential intraoperative and postoperative complications with early postoperative graft detachment being the most common one [5]. Graft detachment vary greatly between studies ranging between 0.9 to 36.4% in studies that include complex cases [6]. Several factors have been reported to be associated with graft detachment in DSAEK including previous failed PKP [6], prior glaucoma surgery [6, 7], compromised iris-lens diaphragm [8], combined phacoemulsification cataract surgery [9] and residual graft-host interface fluid [10].

Intraoperative optical coherence tomography (iOCT) has been shown to be feasible for numerous anterior and posterior segment ophthalmic procedures [11–14]. Specifically, iOCT has been reported to be beneficial during DSAEK procedures and identification of remnant interface fluid [15, 16]. In 2017 Hallahan et al. reported that larger interface fluid volume, area, and thickness at the end of iOCT guided DSAEK were associated with early graft detachment [10].

The aim of this study was to describe and assess outcomes of intraoperative optical coherence tomography (iOCT) guided meticulous peripheral corneal sweeping for removal of interface fluid during ultra-thin DSAEK (UT-DSAEK).

## Methods

### Study participants

This single-center retrospective study included patients who underwent iOCT guided UT-DSAEK from October 2016 to February 2018 at the Department of Ophthalmology of the Hanusch Hospital in Vienna, Austria. Excluded were cases where the preoperative central graft thickness (as measured by the cornea bank) was ≥130 μm or where iOCT video was not of sufficient quality for analysis.

All procedures involving patients were performed in accordance with the Declaration of Helsinki and were approved by the local ethical committee (EK-20-078-VK).

### Data collection

The medical files of all eligible patients were reviewed and the following demographic and preoperative information was collected: age, gender, date of surgery, preoperative CGT and 6 mm diameter graft thickness (Tomey SS-1000 Casia OCT, Tomey Co., Nagoya, Japan) as measured by the cornea bank. The following intraoperative information was collected: CGT after bubbling (first intraoperative measurement), CGT after peripheral corneal sweeping, whether or not venting incisions were performed, presence or remnant interface fluid following sweep and any intraoperative complications. Any postoperative complications including the need for rebubbling were recorded.

### Surgical technique

All surgeries were performed by one experienced surgeon (O.F.) in a similar fashion under neuroleptic anesthesia with sub-tenon anesthesia. The UT-DSAEK grafts were supplied by Fondazione Banca degli Occhi del Veneto Onlus, Zelarino, Venice, Italy and were precut and prestamped (“F”). The partial thickness graft was cut with a punch trephine (8 to 8.5 mm). Ink-marked calipers were used to mark the corneal diameter of the descemetorhexis. A 4.5-mm limbal incision and 3 paracenteses were created and an anterior chamber maintainer was placed. Descemetorhexis was performed using a reverse Sinskey hook. The grafts were inserted into the eye using a Busin glide under the continuous flow of an anterior chamber maintainer. After unfolding and centration a small air bubble was injected to preserve graft positioning and the anterior chamber maintainer was removed and a single 10–0 nylon suture was used to suture the main wound and any other leaking wounds. The anterior chamber was then completely filled with air and centripetal corneal sweeps all the way to the periphery beyond the limbus with a phako-spatula were performed. If remnant interface fluid persisted despite meticulous sweeping then venting incisions were performed at the surgeon’s discretion. The eye was kept with a 100% air fill and slightly above physiological pressure (according to surgeon touch) and topical cyclopentolate 1% drops were instilled to achieve pupil dilation.

### Intraoperative OCT imaging

During the procedure, continuous iOCT imaging with the two-line HD setting, with an axial resolution of 5.5 μm and a transversal resolution of 15 μm, was performed with a commercially available iOCT device (RESCAN 700; Carl Zeiss Meditec AG, Germany). To be able to identify areas of remnant interface fluid the foot pedal was used to actively move the iOCT scans to follow the area of interest. All continuous measurements (videos) were analyzed after screenshots were taken at the timepoints of interest. To measure the distances in pixels within the scans, all screenshots were imported into Photoshop CS6 (Adobe Systems, Inc.) and the values were then converted into millimeters. Analysis of images from the continuous iOCT videos was performed by one examiner to ensure standardized analysis for all patients.

### Statistical analysis

Data were analyzed with the Minitab Software, version 17 (Minitab Inc., State College, PA). For within group analysis of continuous variables the paired t-test was used. For comparison between of continuous and categorical variables between groups the Mann-Whitney test and Chi-Square test were used respectively. In all analyses, a two-sided *P* value < 0.05 was considered statistically significant. All presented means are accompanied by their respective standard deviations.

## Results

Overall, 28 eyes of 28 patients with a mean age 73.9 ± 10.0 years (range 56 to 92 years) of which 25% (*n* = 7) were of male gender were included in this study. No cases were excluded due to CGT ≥ 130 μm or iOCT video being of insufficient quality. Indications for UT-DSAEK were Fuchs endothelial dystrophy (17/28, 60.7%) and pseudophakic bullous keratopathy (11/28, 39.3%). There were no serious intraoperative complications.

### Meticulous peripheral sweeping

**Fig. 1:**
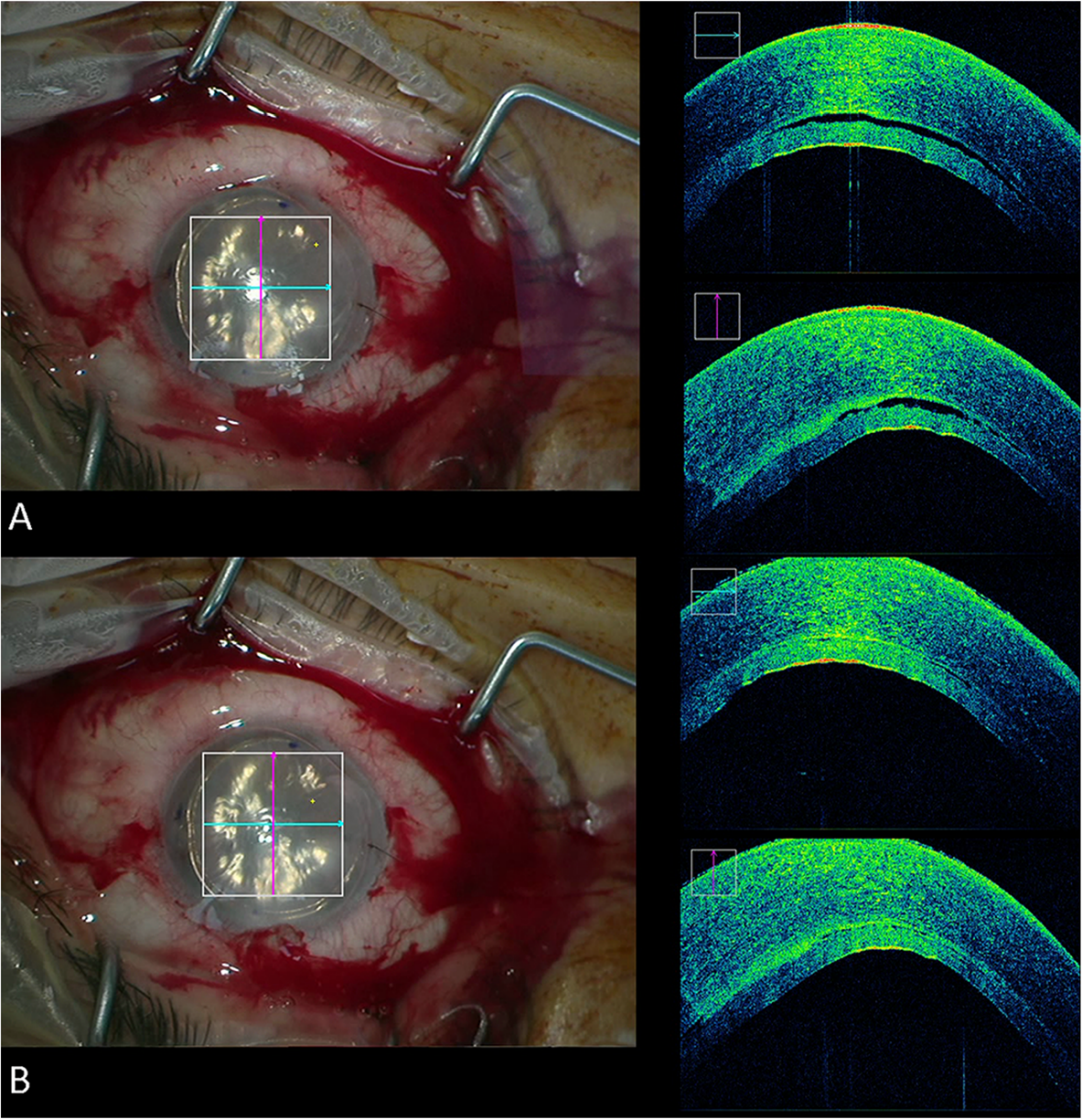
The intraoperative OCT of patient #14 demonstrating remnant interface fluid (**a**) that no longer appears following meticulous peripheral sweeping (**b**)

**Fig. 2:**
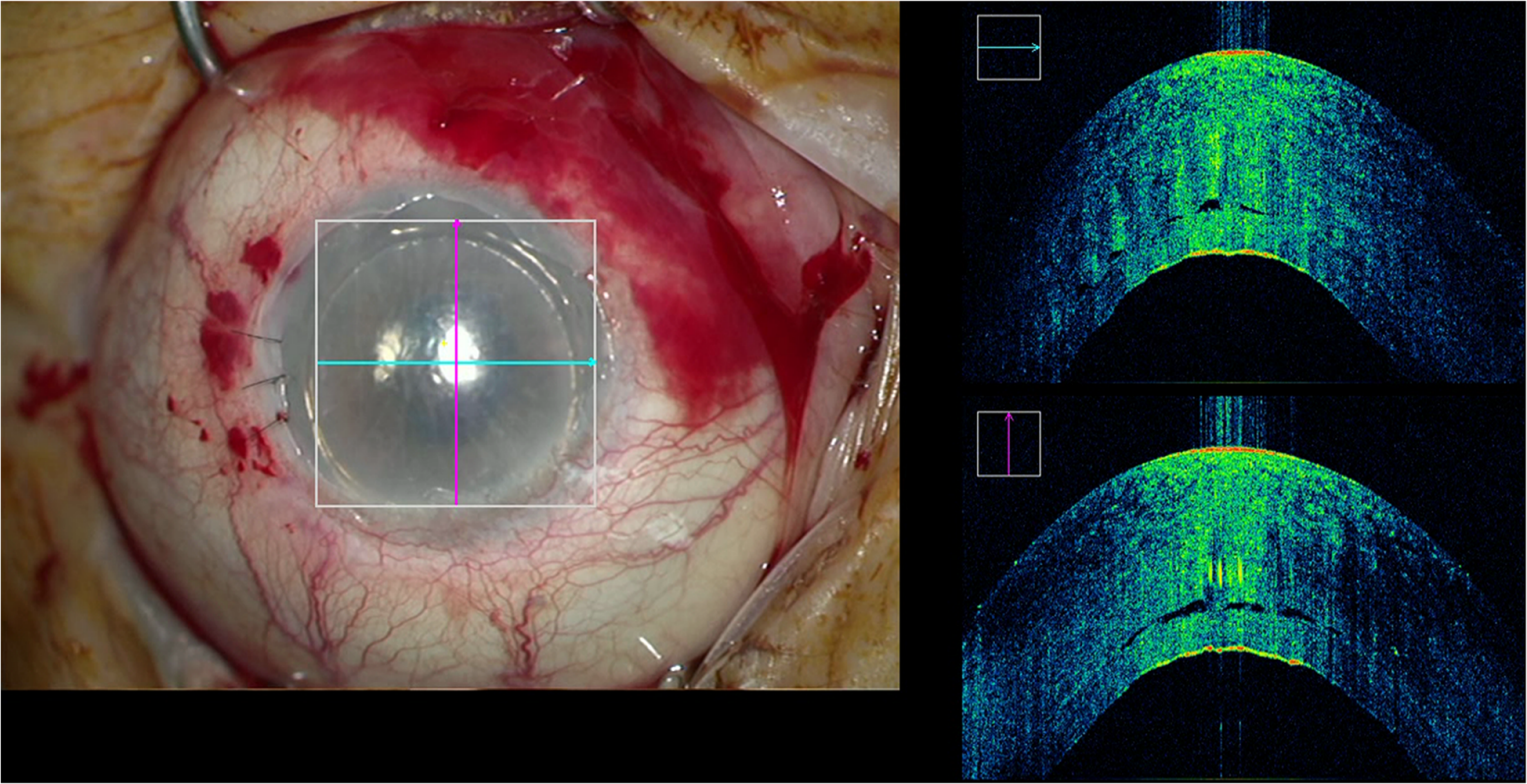
The intraoperative OCT of patient #21, where despite peripheral meticulous sweeping a minute amount of fluid is left in the interface. This patient required subsequent rebubbling during the postoperative follow-up


Meticulous peripheral sweeping was deemed necessary by the surgeon in 89.3% (*n* = 25) of the cases due to remnant fluid identified in the graft host interface (video 1). Following the peripheral sweeping performed, remnant fluid was no longer identified with iOCT in 84% (*n* = 21) (Fig. 1), with a minute amount of interface fluid left in the central 3 mm in 4 cases (16%) (Fig. 2). Venting was performed in one case to remove a substantial amounts of remnant fluid.

**Additional file 1: Video 1.** The intraoperative OCT recording of patient #14 where remnant fluid is left at the graft host interface (undetectable clinically). Following meticulous peripheral sweeping all interface fluid is removed.

### Central graft thickness

**Fig. 3:**
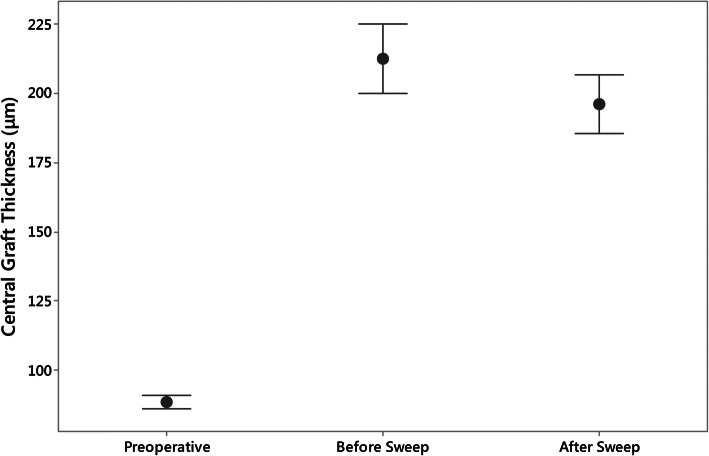
The central graft thickness as measured by the cornea bank, prior to meticulous peripheral sweeping and following sweeping. The graft thickness is significantly increased during surgery (*p* < 0.001) and subsequently decreases following the sweeping procedure (*p* < 0.001)

The cornea bank measured preoperative central and 6 mm diameter graft thickness were 88.50 ± 11.9 μm (range 64 to 109 μm) and 114.5 ± 19.0 μm (range 75.9 to 140.8 μm) respectively. Four grafts had a CGT measurement above 100 μm. Prior to sweeping the central graft thickness was significantly thicker compared to the cornea bank thickness measurements taken 2–3 days earlier (212.4 ± 66.4 versus 88.5 ± 11.9 μm, *p* < 0.001). Following peripheral sweeping the interface fluid height was reduced from 17.31 ± 15.96 μm to 3.46 ± 9.52 μm (*p* < 0.001) and central graft thickness was significantly reduced to 196.1 ± 57.2 (*p* < 0.001), a reduction of 7.7% in thickness (Fig. 3).

### Remnant Interface fluid and Rebubbling

Rebubbling was performed in 17.9% (*n* = 5) of the cases. The single case where venting incisions were deemed necessary was one of the rebubbling cases. Patients that underwent subsequent postoperative rebubbling and those that did not were of similar age (*p* = 0.95) and gender (*p* = 0.79) with similar graft thickness at the different stages of surgery (*p* > 0.05 for all) (Table 1). The rebubbling group had a significantly greater proportion of patients that had remnant fluid identified with iOCT at the end of surgery despite meticulous peripheral sweeping (60.0% versus 4.4%, *p* = 0.01). Eyes with any remnant interface fluid despite meticulous peripheral corneal sweeping were at a higher risk for rebubbling (OR = 33.00, 95% CI 2.25–484.45, *p* = 0.005).

**Table 1:** Comparison of the group of patients that did not require rebubbling following intraoperative assisted UT-DSAEK with meticulous peripheral sweeping and those that did. Data presented as either mean ± SD (median) or %

Parameter	No Rebubbling (*n* = 23)	Rebubbling (*n* = 5)	*P-Value
Age (years)	74.0 ± 9.7 (75)	73.8 ± 12.6 (74)	0.95
Gender (%male)	26.1%	20.0%	1.00
Preoperative CGT (cornea bank measured) (μm)	88.2 ± 12.3 (90)	89.8 ± 11.2 (88)	0.88
Preoperative 6 mm GT (cornea bank measured) (μm)	115.0 ± 20.1 (121)	111.9 ± 14.0 (114)	0.53
Pre sweep CGT (μm)	214.8 ± 69.3 (204)	201.6 ± 56.4 (204)	0.81
Post sweep CGT (μm)	198.8 ± 60.1 (198)	183.6 ± 44.4 (204)	0.61
Remnant interface fluid (%)	4.4%	60.0%	0.01

*CGT* central graft thickness, *GT* graft thickness

*Mann-Whitney for continuous variables and Fisher’s exact test for categorical variables

## Discussion

Remnant interface fluid was identified in nearly 90% of the UT-DSAEK cases after instillation of the air bubble leading to subsequent meticulous peripheral corneal sweeping. The meticulous corneal sweeping led to resolution of interface fluid in an overwhelming majority of cases and reduced intraoperative CGT. In addition, there was an association between immediate postoperative rebubbling and remnant interface fluid despite corneal sweeping. This is, to the best of our knowledge, the first study to characterize iOCT guided meticulous peripheral corneal sweeping in UT-DSAEK.

The ability to identify subclinical interface fluid with the aid of iOCT during DSAEK was first described in 2010 by Ide et al. and Knecht et al. [17, 18] A reduction in interface fluid after air bubbling and a further subsequent reduction following venting incisions was described [18]. These were followed by several small case series describing reduction of remnant interface fluid as seen on iOCT following venting incisions [19, 20]. Price et al. described corneal sweeping followed by venting incisions in order to promote graft adherence [21]. Thereafter, Terry et al. reported repeatedly compressing the corneal surface from the center to the periphery to “milk out” any interface fluid during DSAEK in order to avoid venting incisions [22]. The efficiency of avoiding the need for venting incisions during iOCT guided DSAEK corneal sweeping was later demonstrated by the PIONEER study group [10, 15]. Indeed, in the current study, meticulous peripheral corneal sweeping was necessary in nearly 90% of UT-DSAEK cases and completely resolved interface fluid in 84% (*n* = 21/25) with venting incisions deemed necessary in a single case. Similar to DSAEK, it seems as though meticulous iOCT guided corneal peripheral sweeping may obviate the need for venting incisions in UT-DSAEK as well. We found it to be critical to sweep from the center all the way out until beyond the limbus to “milk” as much interface fluid as possible. This was done from the center into all 4 quadrants. Despite this maneuver, 16% (*n* = 4) of the cases had remnant interface fluid. A potential explanation is that when the anterior chamber is filled with air and the eye is rather firm, it may make meticulous sweeping more difficult. For cases with persistent interface fluid, surgeons may consider slightly reducing the pressure of the eye and attempting the sweep again, a technique that was not assessed in the current study.

In the current study the overall rate of rebubbling (17.9%) was on the higher side of what is reported in the literature. Studies reporting DSAEK results where complex cases were included have reported graft detachment rates ranging between 0.9 to 36.4% [6]. We speculate that this is because our threshold for rebubbling is low as many of the patients treated arrive from far away to receive treatment at our tertiary center. A majority (60%) of the grafts that required rebubbling had small amounts of remnant interface fluid despite peripheral corneal sweeping and/or required a venting incision. A small minority (4.4%) of the eyes that did not require rebubbling had remnant interface fluid. These findings are supported by the PIONEER study where larger residual interface fluid volume, area, and thickness at the end of surgery detected with iOCT were associated with early graft non-adherence in DSAEK [10]. They postulated that overall fluid burden may have played a role in non-adherence of grafts. It may also be that unknown factors which led to incomplete resolution of interface fluid following sweeping may have also contributed to subsequent graft detachment. In any event, similar to DSAEK, UT-DSAEK grafts with remnant interface fluid on iOCT are at a higher risk for immediate postoperative graft detachment requiring rebubbling. It is unclear whether or not venting incisions are indeed helpful in these situations as this was performed in only one eye in the current study. Perhaps non-expansile gas such as SF_6_ or C_3_F_8_ may be considered with an emphasis on keeping the eye more pressurized than usual in these cases.

Several studies have evaluated the utility of iOCT in DMEK surgery. These reports demonstrated that iOCT aided in identifying remnants of host Descemet membrane, identifying and facilitating correct graft orientation [23–25]. Recently, Patel et al. reported in a prospective study (*n* = 100) that for novice DMEK surgeons, complication rates and unscrolling times compared favorably with alternative tissue orientation methods while avoiding the need for external markings [25].

Most studies have focused on interface fluid at different stages with a paucity of data available regarding iOCT assessment of DSAEK graft thickness at different stages of surgery. Steverink et al. (*n* = 8) reported an increase in DSAEK graft thickness after insertion into the anterior chamber, however thickness following corneal sweeping was not reported [26]. Indeed, in the current study, CGT was thicker when measured after air bubbling than measured in the cornea bank 2–3 days before surgery. Interestingly, there was a small (7.7%) yet significant reduction following peripheral sweeping. Further prospective comparative studies with larger sample sizes are needed to assess whether the reduction in thickness is a result of the meticulous sweeping or not.

This study has several limitations including its retrospective nature, small sample size and unbalanced groups when comparing the no rebubbling group to the rebubbling group. However, this sample size was sufficient to demonstrate the efficacy of the technique in resolving remnant interface fluid, reduction in intraoperative graft size and association with rebubbling when fluid persists despite sweeping.

## Conclusions

In summary, iOCT identified subclinical remnant fluid in nearly 90% of UT-DSAEK cases. An iOCT guided peripheral corneal sweeping was associated with a reduction in CGT as well as complete resolution of interface fluid in a majority of cases. This pilot study demonstrates that eyes with persistent remnant fluid despite peripheral corneal sweeping during UT-DSAEK are more likely to require subsequent rebubbling.

## Data Availability

The datasets used and/or analysed during the current study are available from the corresponding author on reasonable request.

## Abbreviations

CGT: Central graft thickness
DMEK: Descemet membrane endothelial keratoplasty
DSAEK: Descemet stripping automated endothelial keratoplasty
GT: Graft thickness
iOCT: intraoperative optical coherence tomography
PKP: Penetrating keratoplasty
UT: Ultra-thin
UT-DSAEK: Ultra-thin DSAEK
